# *In planta *production of two peptides of the Classical Swine Fever Virus (CSFV) E2 glycoprotein fused to the coat protein of potato virus X

**DOI:** 10.1186/1472-6750-6-29

**Published:** 2006-06-22

**Authors:** Gianpiero Marconi, Emidio Albertini, Pierluigi Barone, Francesca De Marchis, Chiara Lico, Carla Marusic, Domenico Rutili, Fabio Veronesi, Andrea Porceddu

**Affiliations:** 1 Dipartimento di Biologia vegetale e Biotecnologie Agroambientali e Zootecniche, Università degli Studi di Perugia, Borgo XX Giugno 74, 06121 Perugia, Italy; 2 University of Illinois at Urbana-Champaign, College of Agricultural, Consumer and Environmental Sciences, Department of Crop Sciences, 289 Edward R. Madigan Laboratory, 1201 West Gregory Drive, Urbana, IL 61801, USA; 3 Istituto di Genetica Vegetale, Sezione di Perugia, Via Madonna Alta 130, 06100 Perugia, Italy; 4 ENEA, Centro Ricerche Casaccia, Roma, Italy; 5 Istituto Sperimentale Zooprofilattico dell'Umbria e delle Marche, Sezione di Perugia, Italy

## Abstract

**Background:**

Classical Swine Fever (CSFV) is one of the most important viral infectious diseases affecting wild boars and domestic pigs. The etiological agent of the disease is the CSF virus, a single stranded RNA virus belonging to the family *Flaviviridae*.

All preventive measures in domestic pigs have been focused in interrupting the chain of infection and in avoiding the spread of CSFV within wild boars as well as interrupting transmission from wild boars to domestic pigs. The use of plant based vaccine against CSFV would be advantageous as plant organs can be distributed without the need of particular treatments such as refrigeration and therefore large areas, populated by wild animals, could be easily covered.

**Results:**

We report the *in planta *production of peptides of the classical swine fever (CSF) E2 glycoprotein fused to the coat protein of potato virus X. RT-PCR studies demonstrated that the peptide encoding sequences are correctly retained in the PVX construct after three sequential passage in *Nicotiana benthamiana *plants. Sequence analysis of RT-PCR products confirmed that the epitope coding sequences are replicated with high fidelity during PVX infection. Partially purified virions were able to induce an immune response in rabbits.

**Conclusion:**

Previous reports have demonstrated that E2 synthetic peptides can efficiently induce an immunoprotective response in immunogenized animals. In this work we have showed that E2 peptides can be expressed *in planta *by using a modified PVX vector. These results are particularly promising for designing strategies for disease containment in areas inhabited by wild boars.

## Background

Classical swine fewer (CSF) is an economically important viral infectious disease affecting domestic pigs and wild boars. Infected animals develop severe leukopenia and immunosuppression accompanied by hemorrhagic lesions, with a mortality of about 90% in infected young pigs [1,2]. The etiological agent of the disease is the CSF virus (CSFV syn. Hog cholera), a single-stranded RNA virus belonging to the genus *Pestivirus*, family *Flaviviridae *[3]. The genome of CSFV contains a single open reading frame encoding for a polyprotein of approximately 4,000 aminoacids that is processed into four structural (the nucleocapsid C and three envelope glycoproteins, E^rns ^or gp44/48, E1 or gp33 and E2 or gp55) and several non-structural proteins by virus-encoded and cellular proteases [4].

E2, the most immunogenic envelope glycoprotein, contains four antigenic domains, from A to D [4-6] located within the N-terminal region of the protein. The E2 region between amino acid 690 and 910 contains the sites recognized by an hyperimmune serum in pigs; it includes the TAVSPTTLR motif, which is conserved in all strains of CSFV, but is divergent from other pestiviruses such as bovine viral diarrhea virus (BVDV) and border disease virus (BDV) [4,7].

Transmission of CSFV may be either pig-to-pig or pig-to-wild boar, or it may also occur through infected semen or via an oral route through contaminated fresh, frozen or cured pig meat products [8]. All preventive measures in domestic pigs and in wild boars have been focused to interrupt the chain of infection and to avoid the spread of CSFV within wild boars as well as to interrupt transmission from wild boars to domestic pigs [9,10]. Epidemiological controls are, however, complicated by the lack of diagnostic tools specific for CSFV, mainly due to the high similarity between CSFV and other pestiviruses [11].

The use of plants to produce vaccines is an emerging strategy that may offers several advantages over conventional vaccines [12,13]. Plants are not attacked by animal viruses and therefore plant-based vaccines are safe for animals and can be produced in non-sterile environments. In addition, some plant organs can be transported or distributed over long distances without the need of particular treatments such as refrigeration. Vaccination costs could therefore be dramatically reduced and large areas, populated by wild animals, could be easily covered.

Among the available technologies to express foreign proteins in plants, plant virions represent one of the most advantageous systems for epitope presentation and purification [14]. The macromolecular nature of virion aggregates permits the development of easy procedures for virion purification/concentration: in salty reaction, high doses of peptide can be recovered by a simple centrifugation [15]. Moreover, peptide fused to coat proteins (CP) have been reported to be able to induce a specific immunoglobulin G (IgG) response through a mechanism involving co-stimulatory ability of antigen-presenting cells [14,16].

The demonstration that parenteral administration of synthetic peptides of the E2 is able to induce neutralizing antibodies [4,7,17] has drawn attention to setting up cost effective systems for epitope production. Uhde et al. [18] and Marusic et al. [16] have demonstrated that short peptides (<15 amino acids) can be expressed when directly fused to the CP of potato virus X. In this study we have explored the feasibility of expressing longer peptides (>40 amino acids) of the E2 protein fused to the coat protein of PVX via 2A peptide from FMDV (Foot and Mouth Disease Virus). RT-PCR and sequence analyses demonstrated that this expression system is highly reliable. The potential of such an expression system for vaccination procedures and for enhancing the specificity of diagnostic tools is also discussed.

## Results

### Epitope coding sequence is correctly retained during virus replication

**Figure 1:**
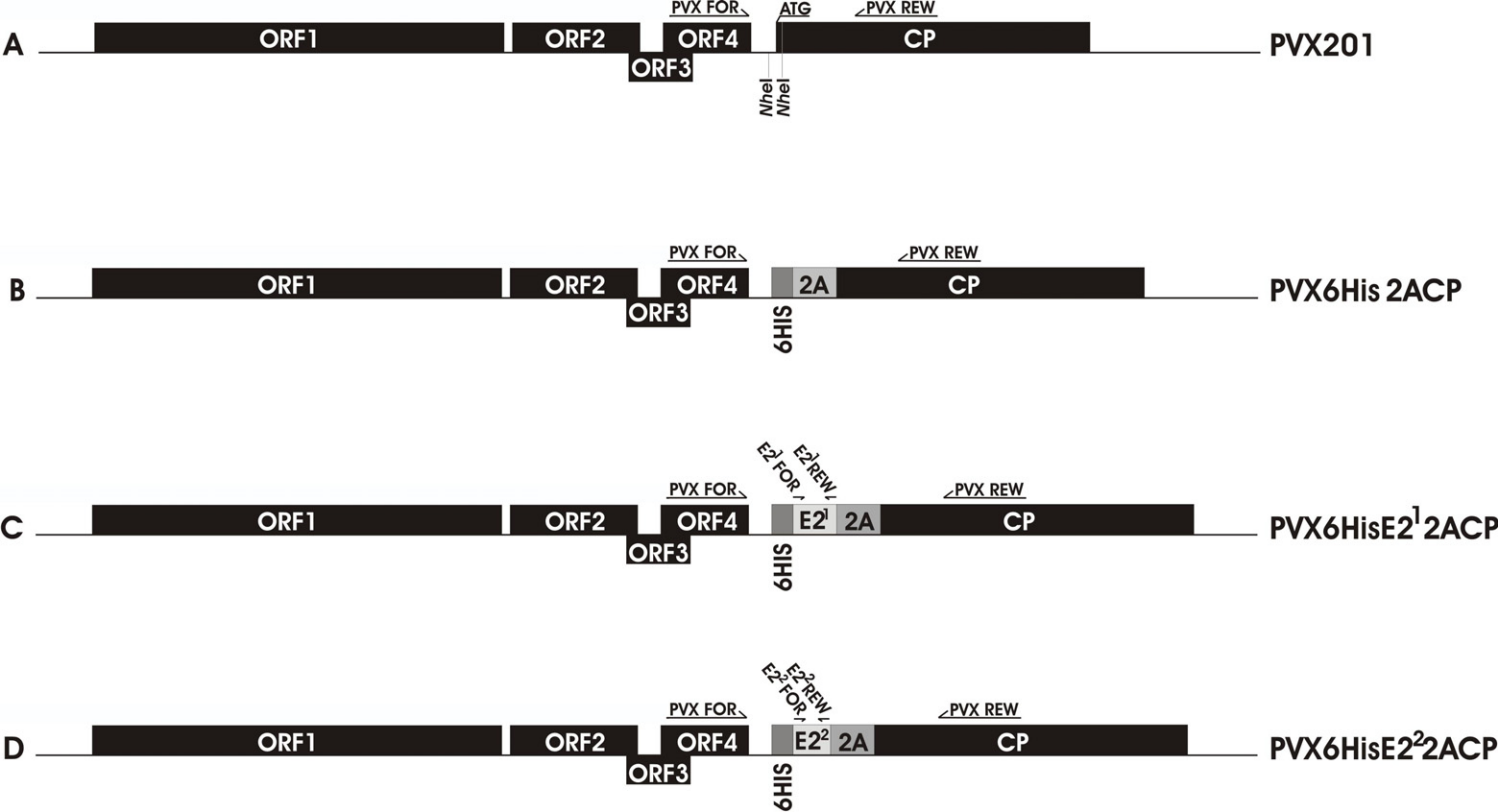
**PVX-derived vectors. **Schematic representation of PVX-derived vectors used. Primers are indicated in their specific annealing sites.

**Figure 2:**
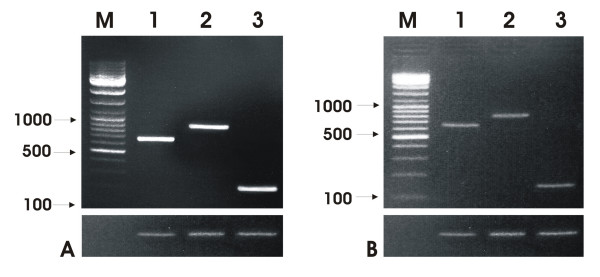
**Expression analyses. **RT-PCR of samples derived from third order infections' systemic leaves; the plants infected with PVX6His2ACP (A1 and B1), PVX6HisE2^1^2ACP (A2) and PVX6HisE2^2^2ACP (B2) vectors amplified with PVX FOR and PVX REW specific primers. Lane A3 shows the RT-PCR results for PVX6HisE2^1^2ACP using E2^1 ^FOR and E2^1 ^REW specific primers, whereas in lane B3 for PVX6HisE2^2^2ACP samples amplified by E2^2 ^FOR and E2^2 ^REW specific primers are shown. To evaluate the relative levels of mRNAs, the *actin *gene was used as an internal control (lower A and B panels). Each experiment was performed in triplicate.

Since the integration of foreign sequences in the PVX genome, or the modification of the CP, may cause genomic rearrangements [18,19], we have verified the integrity of the chimeric transcript. RT-PCR carried out with PVX and epitope specific primers (Fig. 1, Table 1) resulted in a single amplification product of the expected size in cDNA samples derived from plants infected with PVX6HisE2^1^2ACP or PVX6HisE2^2^2ACP plasmids (Fig. 2). No amplification products were observed in samples when the RT step was omitted (data not shown).

Cellular extracts from plants inoculated with plasmid DNAs were used to infect new plants (second order infections) and those obtained from second order infected plants to inoculate other plants (third order infections). Only bands of the expected sizes were visualized in RT-PCR experiments carried out on samples derived from plants of second and third order infections. Figure 2 shows the results of RT-PCR experiments carried out on mRNAs isolated from systemic leaves derived from third order infections. These results demonstrated that the epitope sequences are correctly retained during PVX replication. Direct sequencing of the RT-PCR products obtained from the third-order samples, with PVX specific primers, showed the absence of point mutations (data not shown), leading to the conclusion that epitope coding sequences were replicated with high fidelity during viral infections.

### Both cleaved CP and epitope-CP uncleaved polyproteins are produced upon virus infections

Total soluble proteins, extracted from symptomatic leaves 15 dpi with PVX6His2ACP, PVX6HisE2^1^2ACP, PVX6HisE2^2^2ACP constructs or from mock-infected plants, were analyzed by Western blotting with a polyclonal anti-CP antibody. The conformation of the 2A within the P ribosome site may cause the cleavage of the nascent chain with the subsequent release of the N terminal product from the ribosome. Some ribosomes however will continue to produce the downstream protein as a discrete entity [20,21]. Processing of the 2A site in synthetic polyproteins can be incomplete and the proportion of cleaved versus uncleaved polyprotein may vary depending on the sequence context upstream the 2A site [20].

**Figure 3:**
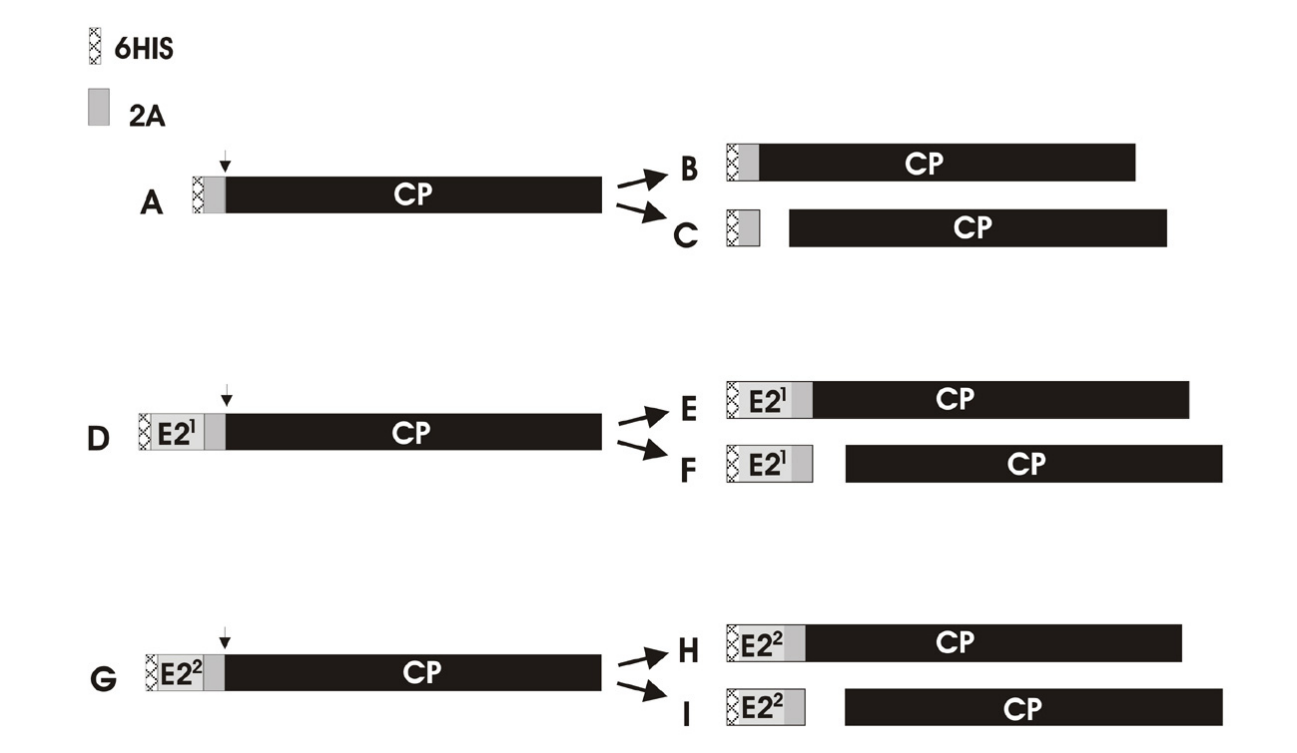
**Putative proteins produced. **Possible translation products of PVX6His2ACP (A), PVX6HisE2^1^2ACP (D) and PVX6HisE2^2^2ACP (G). The small arrows indicate where the polyprotein is expected to cleaved at the carboxyl terminus of the 2A. The PVX6His2ACP messenger (A) will be translated either into an uncleaved polyprotein (B) or into two cleaved subunits: 6His2A and CP, respectively (C). The same holds true for the PVX6HisE2^1^2ACP (D), which could be translated into an uncleaved polyprotein (E) or into two cleaved subunits: 6HisE2^1^2A and CP, respectively (F). Either an uncleaved polyprotein (H) or two cleaved subunits (I) 6HisE2^2^2A and CP are expected to be translated from PVX6HisE2^2^2ACP (G).

**Figure 4:**
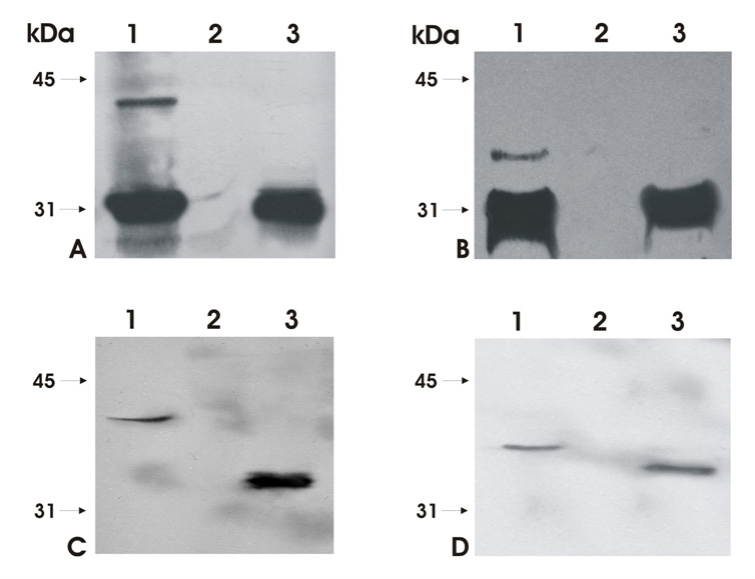
**Both uncleaved and cleaved CP protein are produced. **Western blotting analysis carried out on protein extracts using a rabbit polyclonal anti-CP antibody (A and B) and a mouse monoclonal anti-6His antibody (C and D). Fifteen micrograms of protein were prepared from mock-inoculated control plants (A2, B2, C2 and D2), plants inoculated with PVX6His2ACP (A3, B3, C3 and D3), plants inoculated with PVX6HisE2^1^2ACP (A1 and C1) or with PVX6HisE2^2^2ACP (B1 and D1). Standards Molecular weight are shown on the left of each figure (kDa = kiloDaltons).

Translation of chimeric transcripts is therefore expected to produce: i) a full length uncleaved polyprotein (Fig. 3), ii) the N-terminal cleaved portion of the polyprotein and iii) the CP cleaved protein corresponding to the C-terminal portion of the polyprotein (Fig. 3). A single broad band of about 31–34 kDa corresponding to the cleaved CP and to the uncleaved 6His2ACP polyproteins, respectively, were visualized in PVX6His2ACP samples (Fig. 4 A3 and B3). Accordingly, in Western blotting of *N. benthamiana *infected with a similar PVX2A vector, Santa Cruz et al. [22], detected the cleaved CP protein at 31 kDa.

In PVX6HisE2^1^2ACP-infected samples, two bands of about 31 and 42 kDa were visualized (Fig. 4 A1). These bands likely correspond to the cleaved CP protein and to the uncleaved 6HisE2^1^2ACP protein, respectively (Fig. 3A–C). In PVX6HisE2^2^2ACP-infected samples, the 37 and 31 kDa bands (Fig. 4 B1) correspond to the uncleaved 6HisE2^2^2ACP polyprotein and to the cleaved CP protein, respectively (Fig. 3D–F). No bands were visualized in the sample of mock infected plants (Fig. 4 A2, B2, C2 and D2).

Duplicate blots were also probed with an anti-6His antibody. Only a single band of 42 kDa and 37 kDa were visualized in PVX6HisE2^1^2ACP and PVX6HisE2^2^2ACP-infected samples (Fig. 4 C1 and D1). While a signal of about 34 kDa was visualized in PVX6His2ACP infected plants (Fig. 4 C3 and D3), no signal was detected in uninfected plants (Fig. 4 C2 and D2). Bands of about 11 and 6 kDa, corresponding to the predicted molecular weights for 6HisE2^1^2A and 6HisE2^2^2A were never observed. These results demonstrate that the PVX6His-recombinant vectors are able to drive the expression of an uncleaved 6His-epitope-2ACP polyprotein.

### Recombinant virions can be partially purified by centrifugation

**Figure 5:**
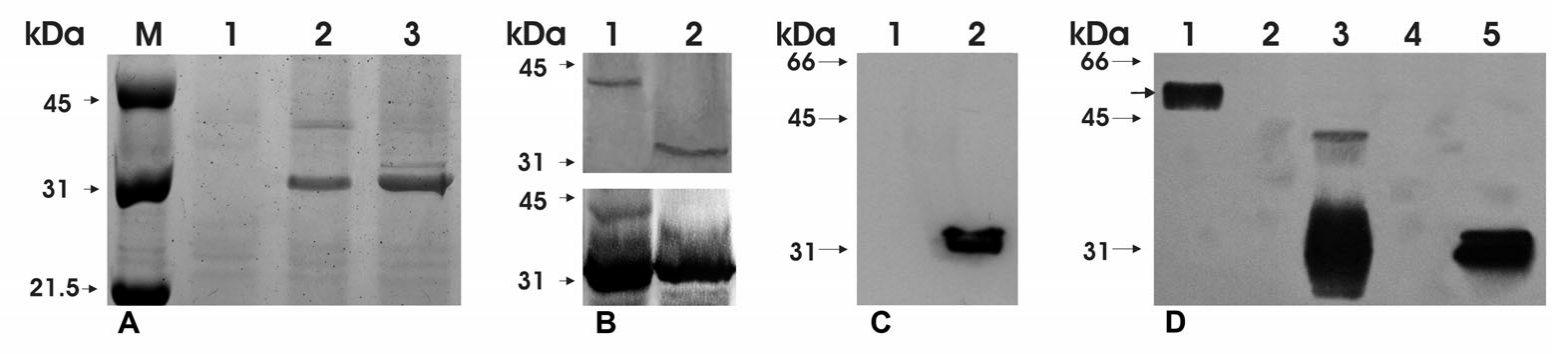
**Purified virions are immunogenic. **Staining with Comassie Brilliant Blue (A) of mock-inoculated control plants (A1), partially purified viral particles of the PVX6HisE2^1^2ACP (A2) and PVX6His2ACP (A3). Western blotting with anti-6His (B upper) and anti-CP (B lower) antibodies of partially purified viral particles of the PVX6HisE2^1^2ACP (B1) and PVX6His2ACP (B2). Western blotting analysis (C) using sera from rabbit inoculated with PVX6His2ACP against proteins purified from mock-inoculated control plants (C1) and from PVX6His2ACP (C2). Western blotting analysis (D) using sera from rabbit inoculated with PVX6HisE2^1^2ACP. Purified E2 protein (D1; E2 control protein is indicated with a bold arrow), marker (D2), proteins purified from plants inoculated with PVX6HisE2^1^2ACP (D3), mock-inoculated control plants (D4) and PVX6His2ACP (D5). Standards Molecular weight (kDa = kiloDaltons) are shown on the left of each figure.

Recombinant virions were partially purified by centrifugation of cellular extracts from PVX6His2ACP and PVX6HisE2^1^2ACP infected plants after incubation on ice for 2 h in presence of 8% PEG-8000 as reported by AbouHaidar et al. [23]. The purification yielded about 150 and 120 μg of virus particles per gram of leaves infected with PVX6His2ACP and PVX6HisE2^1^2ACP, respectively. An aliquot of 250 ng of purified protein for each construct was fractionated by SDS-PAGE and stained with Comassie Brilliant Blue. Two bands of 42 and 31 kDa were visualized over a very weak background (Fig. 5A). The 42 kDa:31 kDa relative amount, quantified with GeneSnap software (Syngene), was 1:2. Western blotting with anti-6His (Fig. 5B upper) and anti-CP (Fig. 5B lower) antibodies confirmed that these bands correspond to the uncleaved 6HisE2^1^2ACP polyprotein and to cleaved CP protein.

### Epitope E2^1 ^fused to the PVX CP protein is immunogenic

**Figure 6:**
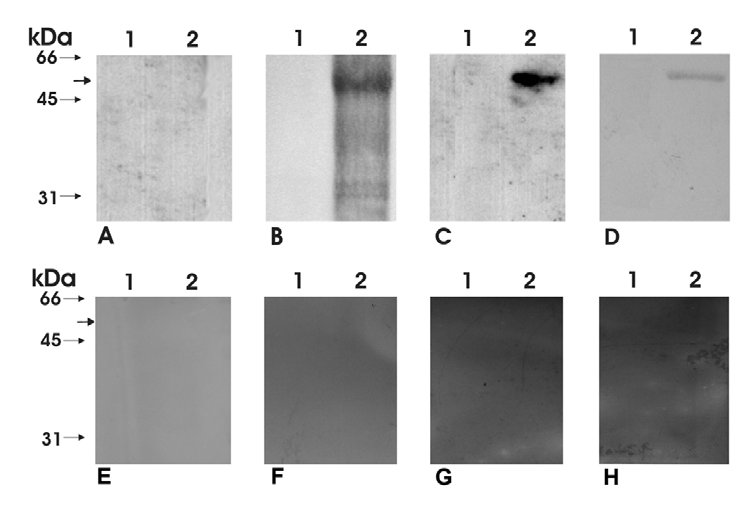
**Sera from immunized rabbits contain anti-E2-specific antibodies. **Pre-immune sera (A and E) and sera from rabbit immunized with PVX6HisE2^1^2ACP (B, C, D) and with PVX6His2ACP (F, G, H) were tested by Western blotting for antibody reactivity against E2 (indicated with a bold arrow) protein (lanes A2, B2, C2, D2, E2, F2, G2 and H2). Western blotting was carried out using serial dilutions 1/10 (B and F), 1/100 (C and G), 1/1,000 (D and H) and pre-immune sera 1/100 (A and E). Lanes A1, B1, C1, D1, E1, F1, G1, and H1 represents Standards Molecular weight.

To verify that the epitopes produced *in planta *are immunogenic, rabbits were immunized with 50 μg of purified PVX6HisE2^1^2ACP virions (group A) or with purified PVX6His2ACP (group C). The presence of anti-E2 antibodies in rabbit sera was tested by Western analyses of purified E2 proteins. Western blotting tests were performed with serial dilutions of sera from rabbit immunized with PVX6HisE2^1^2ACP (Fig. 6A–D) and PVX6His2ACP (Fig. 6E–H). The E2 specific band was detected with a PVX6HisE2^1^2ACP sera dilution up to 1/100 (Fig. 6A–D) but not with PVX6His2ACP at any dilution (Fig. 6E–H). To verify the immunogenic response of rabbits inoculated with PVX6His2ACP proteins (Fig. 5C), western blotting analysis were carried out using their sera against proteins purified from mock-inoculated control plants (Fig. 5 C1) and from plants infected with PVX6His2ACP (Fig. 5 C2). As shown in Figure 5D, a 55 kDa band, corresponding to the E2 protein, was visualized in blots probed with rabbit sera but not in duplicate blots probed with pre-immune sera or sera bled from B and C group rabbits (data not shown). The A group rabbit sera recognized both the 42 kDa and 31 kDa proteins in total protein extracts derived from PVX6HisE2^1^2ACP-infected plants (Fig. 5D). These results suggest that the PVX6HisE2^1^2ACP antibody is able to induce anti-E2 antibodies in immunized rabbits.

## Discussion

Plant virus-mediated expression of immunogenic peptides gives several advantages over other available systems for mass production of peptides including: i) the environments for virus infection require only a moderate sterility (a phyto-pathogen proof greenhouse would be sufficient), ii) the need for "cold chains" from the producer to the site of use of the vaccine may, in some cases, be avoided for plant-derived vaccines [12] and iii) high doses of peptide can be easily recovered from infected tissues using basic laboratory equipment.

However, the reliability of this expression system can be occasionally undermined due to several factors. First, viral vectors have the tendency to lose the inserted sequences after a number of replication rounds or plant passages [14]. Second, a high error frequency has been observed during viral RNA synthesis [24]. Detailed analysis of the reliability of the expression levels achievable with these systems is therefore advisable before large scale applications. Avesani et al. [25] have observed a drastic decline of GAD 65, PVX-mediated expression levels after the first passage in *N. benthamiana *plants and Ziegler et al. [19] have shown that a scFv sequence inserted in a PVX vector is completely deleted after the first round of infection. Similar results were recently reported by Uhde et al. [18] using PVX-based vectors.

In the present study, we have shown that a modified PVX viral vector can be successfully used to express CSFV peptides (70 aa for E2^1 ^and 40 aa for E2^2^) linked to the N-terminus of the PVX coat protein without compromising the stability of virus during generations. Expression and sequence analyses, carried out for three successive infection cycles, demonstrated that this expression system is highly reliable. Moreover, rabbit immunization experiments showed that recombinant virions are effective in inducing antibodies.

Two factors may have contributed to the high stability of the PVX construct used in this work: i) the presence of a single subgenomic CP promoter may have reduced the possibility for homologous recombination [26] and ii) the co-presence of free and uncleaved CP protein may have preserved an acceptable level of virus infectivity [22]. The other important advantage of the PVX expression system is related to the co-stimulatory upregulation achieved by peptides linked to the PVX coat protein. Since this effect has been demonstrated for two independent proteins, namely scFv [27] and HIV [16], it is possible that this reflects a feature inherent to the PVX coat protein or, alternatively, it could be due to the form in which it is presented.

Legocki et al. [28] have demonstrated that an immunoprotective response is induced in mice by oral administration of E2 glycoprotein produced *in planta*. Epitope mapping studies have begun to identify the minimal immunogenic site of various monoclonal or polyclonal antibodies directed against the E2 protein. The mass production of specific E2 peptides will be needed to improve the specificity of diagnostic kits and to induce highly specific immunization. Owing to the high speed of plant viral expression systems and to the ease of recovery of PVX recombinant viral particles, we believe that the expression system described could be extremely useful.

Recently, Uhde et al. [18] and Marusic et al. [16] have demonstrated that small peptides shorter than 15 amino acids can be expressed if directly linked to the N-terminus of the PVX-CP. Other studies have shown that with the 2A-based overcoating strategy, the PVX can be used as a presentation system for proteins or peptides such as green fluorescent protein [22], the highly conserved ELDKWA peptide from glycoprotein 41 of scFv [29] and rotavirus major capsid protein (VP6) [30] but no direct assessment of insert stability was reported. Our study demonstrated that the 2A based overcoating strategy allows to express peptides long up to 70 aa without a significant loss of insert.

Further studies are required to verify whether a size constrain on insert length exists. In addition, other factors related to insert sequence may further influence insert stability. For example, the presence of recombinationally active sequence (RAS), i.e regions of homology between the insert and part of the vector or the formation of local double stranded regions within the insert sequence were demonstrated to promote heterologous recombination [31].

Our ongoing experiments are aimed towards optimization of two-step purification procedures that will allow both recovery of recombinant virions by PEG precipitation and purification of the uncleaved 6His-epitope-CP polyprotein by anti 6His affinity chromatography.

Finally, concerns may be raised regarding the safety of this expression system in regions where either tobacco or potato are widely cultivated. In addressing this issue, it is useful to stress that there is no evidence that naturally occurring vectors (insect, nematodes or fungal) transmit PVX. Moreover, the virus is not transmitted through seed or pollen and thus the risk of its accidental release into the environment is exceedingly small. Therefore, environmentally safe conditions could be easily obtained with a moderate investment in infrastructures.

## Methods

### Double stranded oligolinkers

To express peptides fused to PVX coat protein (CP), an in-frame sequence encoding 16 amino acids of the 2A oligopeptide (NFDLLKLAGDVESNPG) of the Foot-and Mouth Disease Virus (FMDV) [32], was cloned between the 3' end of a sequence encoding for a 6 Histidine tag (6His) and the fourth codon of the coat protein coding sequence (CDS) (Fig. 1). Transcription of the chimeric messenger was driven by a duplicated CP promoter of PVX [33]. The double-stranded 2A oligolinker was prepared by mixing 500 pmol of the 2A upper (5'-TCGACAATTTTGATTTATTAAAGTTAGCAGGTGATGTTGAATACAATCCAGGTCCAG-3') and 2A lower (5'-CTGGACCTGGATTTGATTCAACATCACCTGCTAACTTTAATAAATCAAAATTGTCGA-3') oligos. The 6His-tag double stranded oligolinker was prepared by mixing 500 pmol of 6His upper (5'-TCGAGATGCACCACCACCACCACCACG-3') and 6His lower (5'-TCGACGTGGTGGTGGTGGTGGTGCATC-3') oligos. The oligonucleotide mixtures were heated at 95°C for 10 min and then cooled at room temperature to allow annealing.

### Construction of PVX6His2ACP vector

The PVX201 vector (kindly provided by David Baulcombe, The Sainsbury Laboratory, Norwich, UK, [34]) which contains the PVX cDNA under the transcriptional control of the Cauliflower 35S promoter and the nopaline synthase terminator [33] was digested with *Nhe*I (New England Biolabs) and then blunt-ended by Klenow polymerase (New England Biolabs). The linearized vector was then ligated to the 2A double stranded oligolinker to produce the PVX2ACP vector.

The PVX2ACP vector was then linearized with *Sal*I and ligated to the 6His-tag oligolinker to produce the PVX6His2ACP vector. This vector contains a unique *Sal*I site to be used for cloning coding sequences for the gene of interest that is fused in frame to the 3' terminus of the 6His-tag and to the 5' end of the 2A sequence.

### Construction of PVX6HisE2^1^2ACP and PVX6HisE222ACP

The E2^1 ^(region between amino acids 790 and 860) and E2^2 ^(region between amino acids 854 and 894) sequences of the E2 protein were amplified by PCR using specific primers (Table 1) that contained a *Sal*I site to allow subsequent cloning into PVX6His2ACP.

The amplification fragments were cloned into a PCR4 TOPO vector using the TOPO TA cloning for sequencing kit (Invitrogen) and sequenced to verify the absence of point mutations. The E2^1 ^and E2^2 ^fragments were excised from the respective PCR4 vectors using *Sal*I and separated on a 1% agarose gel. The bands were then purified from the gel using the GFX™ PCR DNA and Gel Band Purification kit (Amersham Biosciences).

The PVX6His2ACP vector, linearized with *Sal*I and dephosphorylated using calf intestinal alkaline phosphatase (New England Biolabs), was ligated to either the E2^1 ^or E2^2 ^fragments to produce PVX6HisE2^1^2ACP and PVX6HisE2^2^2ACP, respectively.

### Plant infection and RT-PCR analysis

*Nicotiana benthamiana *plants were grown at 22°C with a day-light cycle of 16 h of light and 8 h of dark. Two healthy leaves per plant were infected using 20 μg of either PVX6His2ACP, PVX6HisE2^1^2ACP or PVX6HisE2^2^2ACP plasmid as described by Marusic et al. [16].

Total RNAs were isolated from symptomatic leaves two weeks after the infection using the GenElute Mammalian Total RNA miniprep kit (Sigma) according to the manufacturer's instructions. Total RNA was purified from residual genomic DNA using the DNA-free kit (Ambion). First strand cDNA was synthesized from 1 μg of total RNA in a volume of 50 μl according to Sambrook and Russell [35]. RT-PCR reactions were performed in a total volume of 50 μl containing 1 μl of first-strand cDNA, 1 μM of each primer, 1× PCR buffer, 1.5 mM MgCl_2_, 0.2 μM dNTPs and 2 U of recombinant Taq DNA polymerase (Invitrogen). The DNA was denatured at 94°C for 1 min, and then subjected to 25 cycles of 94°C for 1 min, Tm (specific for each primer pair, Table 1) for 1 min and 72°C for 1 min, plus a final extension at 72°C for 10 min. Table 1 gives the sequence of primers used for RT-PCR experiments (Fig. 2). Sequencing was performed using an ABI Prism 377 (Applied Biosystems) and the ABI Prism Big Dye Terminator v1.1 Ready Reaction Cycle Sequencing kit (Applied Biosystems). Primers used for sequencing reactions were PVX FOR and PVX REW (Table 1).

### Protein extraction and quantification

*N. benthamiana *leaves were ground to a fine powder in liquid nitrogen. Approximately 0.5 g of leaf powder was resuspended in 0.7 ml of extraction buffer (50 mM Tris-HCl, 150 mM NaCl, 1 mM PMSF, 0.2% Triton X-100, pH 8). The homogenates were centrifuged at 15,000 × g at 4°C for 20 min and the supernatants were quantified using the Bradford protein assay kit (Bio-Rad) using Bovine Serum Albumin (BSA) as reference standard.

### SDS/PAGE and Western blotting analysis

Equal amounts of total soluble proteins have been loaded on 12% SDS-polyacrylamide gels according to Sambrook and Russell [35]. Gels were either stained with Coomassie Brilliant Blue (Bio-Rad) according to Sambrook and Russell [35] or transferred to a Hybond C^+ ^membrane (Amersham) using a semidry blotting apparatus (Bio-Rad) at 20 V for 12 h at 4°C. The membranes were blocked with 10% non-fat dry milk (Bio-Rad) in T-TBS for 1 h at room temperature. A 1: 10,000 rabbit polyclonal anti-CP antibody (1:10,000; kindly provided by CNR-IVV) and an anti-rabbit IgG peroxidase conjugated (1:5,000; Sigma) were used as primary and secondary antibodies, respectively. A mouse monoclonal anti-6His antibody (1:3,000; BD Biosciences) and an anti-mouse peroxidase conjugate (1:10,000; Sigma) were used for Western blotting. Hybridized blots were exposed with Kodak AR films (Kodak) after the chemiluminescent reaction carried out using the ECL kit (Pierce).

### Partial purification of PVX particles

Virus particles were purified from plant leaves infected with PVX6His2ACP or PVX6HisE2^1^2ACP according to a modified method of AbouHaidar et al. [23]. Briefly, powdered infected leaves (5 g) were suspended in 20 ml Tris-borate buffer containing 0.2% β-mercaptoethanol. The suspension was filtered through four layers of cheesecloth and n-butanol was added to a final concentration of 6%. The mixture was kept on ice for 2 h with constant stirring and then centrifuged for 10 min at 15,000 × g. PEG 8,000 was added to a final concentration of 8% in the presence of 2% NaCl on ice for 2 h with constant stirring, decanted for another 2 h, and then centrifuged at 15,000 × g for 10 min. The pellet was resuspended in Tris-borate buffer, pH 7.5 at 4°C overnight. The virus solution was then centrifuged three times at 7,500 × g for 5 min each. After precipitation, particles were directly dialyzed against 1× PBS. The concentration of virus was calculated using the PVX extinction coefficient (2.97) at 260 nm as reported by Uhde et al. [18].

### Rabbit immunizations

Three months old rabbits (New Zealand Breed) were divided in groups A, B and C of four rabbits each and injected by subcutaneous route using 50 μg of purified proteins containing Freund's adjuvant. Immunizations were repeated after 21 and 45 days without adjuvants. The A group was immunized with PVX6HisE2^1^2ACP, the B group with PBS only and C group with PVX6His2ACP purified virions. Fifteen days after the last immunization, rabbits were bled and serum was recovered. Preimmune sera (Fig 6A and 6E) and sera dilutions (1/10, 1/100, 1/1000) from A and C groups (Fig. 6B–D and 6F–H) were then tested for antibody reactivity against E2 following standard procedure. E2 protein was specifically recognized only by sera from A group rabbit dilutions up to 1/100 (Fig. 6B–D). Baculovirus produced E2 protein, kindly provided by the Italian Reference Centre for Classical Swine Fever (Istituto Zooprofilattico dell'Umbria e delle Marche), was resuspended in sample buffer and loaded in 12% SDS-PAGE and blotted to Hybond C^+ ^membrane (Amersham).

## Authors' contributions

GM, PB, FDM have carried out most of the cloning work and expression analysis. CL, CM have carried out the virions purification. AP and EA have coordinated the group and contributed to the development of the experimental strategy. DR has immugenized rabbits. FV was the responsible for the research program.

## Figures and Tables

**Table 1:** Primer sequences. PVX and epitope-specific primers, including primer sequences and amplification product characteristics for PVX6His2ACP (A), PVX6HisE2^1^2ACP (B) and PVX6HisE2^2^2ACP (C).

Name	Primer sequence 5' to 3'	Optimal annealing temp. °C	Amplified fragment size (bp)		
			A	B	C
PVX FOR PVX REW	CTGGGGAATCAATCACAGTG GTCGAATGCAGCGAATTTGTGC	61.5	677	890	800
E2^1 ^FOR E2^1^REW	GTCGACGGGCTGTGCCCATTTGATACG GTCGACACTGTTGTGGTCACACAATCC	63	--	213	--
E2^2 ^FOR E2^2^REW	GTCGACATGGATTGTGTGACCACAACA GTCGACTACTAGCCCCCCCGTGTAGAC	64	--	--	123
